# Epidemiologic Features of Kawasaki Disease in Shanghai From 2013 Through 2017

**DOI:** 10.2188/jea.JE20190065

**Published:** 2020-10-05

**Authors:** Li-ping Xie, Wei-li Yan, Min Huang, Mei-rong Huang, Sun Chen, Guo-ying Huang, Fang Liu

**Affiliations:** 1 Heart Center, Children’s Hospital of Fudan University, Shanghai, China; 2 Department of Clinical Epidemiology, Children’s Hospital of Fudan University, Shanghai, China; 3 Department of Cardiology, Shanghai Children’s Hospital, Shanghai Jiaotong University, Shanghai, China; 4 Pediatric Heart Center, Shanghai Children’s Medical Center, Shanghai, China; 5 Department of Pediatric Cardiology, Xinhua Hospital, Affiliated to Shanghai Jiao Tong University School of Medicine, Shanghai, China

**Keywords:** Kawasaki disease, epidemiology, coronary artery lesion, Shanghai

## Abstract

**Background:**

We sought to investigate epidemiologic features of Kawasaki disease (KD) in Shanghai from 2013 through 2017 and identify risk factors for coronary artery lesions (CAL).

**Methods:**

As in our previous three surveys, a set of questionnaires and diagnostic guidelines for KD were sent to 50 hospitals providing pediatric medical care in Shanghai. Medical records of KD patients diagnosed from January 2013 through December 2017 were retrospectively analyzed. Multivariate logistic regression analysis was performed to identify risk factors for CAL.

**Results:**

A total of 4,452 cases were enrolled. Male-to-female ratio was 1.7:1. The incidence of KD was 68.8 to 107.3 per 100,000 children aged <5 years from 2013 to 2017. Age at onset ranged from 15 days to 14.0 years (median: 1.8 years). KD occurred more frequently in spring and summer. Of 4,325 patients (97.0%) receiving intravenous immunoglobulin (IVIG), 362 (8.4%) were resistant to initial IVIG. CAL occurred in 406 (9.1%) patients, including 118 (2.7%) with medium aneurysms and 31 (0.7%) with giant aneurysms. Recurrent cases were 60 (1.3%). No death was found in this survey. Higher platelet levels, lower albumin levels, male sex, incomplete KD, IVIG resistance, and receiving initial IVIG ≤4 days or >10 days, were independently associated with CAL.

**Conclusions:**

The incidence of KD in Shanghai had substantially increased while the proportion of CAL had substantially decreased as compared with our previous surveys. Higher platelet levels, lower albumin levels, male sex, incomplete KD, IVIG resistance, and receiving initial IVIG ≤4 days or >10 days, were risk factors for CAL.

## INTRODUCTION

Kawasaki disease (KD) is an acute, self-limited febrile illness that predominantly affects children under 5 years of age. The etiology remains unclear. It is a systemic vasculitis mainly affecting coronary arteries^1^ and is becoming the leading cause of childhood acquired heart disease both in developed countries and China.^2^

The incidence of KD remains highest in Japan,^3^ followed by South Korea,^4^ Taiwan,^5^ and mainland China,^6^^,^^7^ whereas it was much lower in European^8^ and American countries.^9^^,^^10^ Actually, the incidence of KD has recently shown an increasing trend in many regions, especially in Asian countries.^3^^–^^5^^,^^7^ In Shanghai, a well-developed city in China, the incidence of KD increased from 16.8 to 55.5 per 100,000 children <5 years old from 1998 through 2012.^6^^,^^11^

Coronary artery lesions (CAL) are still the main factors affecting the long-term prognosis and mortality of KD. Fortunately, the proportion of CAL among KD patients showed a decreasing trend with the increasing administration of large dose of intravenous immunoglobulin (IVIG).^3^^,^^4^^,^^8^^,^^9^ In Shanghai, the proportion of CAL decreased from 19.8% to 15.9% from 1998 through 2012.^6^^,^^11^

We have conducted three epidemiologic surveys of KD in Shanghai since 1998 (once every 5 years). Considering that epidemiologic features of KD keeps changing since our first survey, especially the incidence of KD and the proportion of CAL, we continued to carry out the fourth survey to keep track of the latest changes. We aimed to provide the descriptive epidemiology of KD in Shanghai from 2013 through 2017 and identify independent risk factors for CAL.

## MATERIALS AND METHODS

### Data collection

As in our previous three surveys, a set of questionnaires and diagnostic guidelines were sent to all hospitals providing pediatric medical care in Shanghai (50 in total). All patients discharged from these hospitals from January 1, 2013 through December 31, 2017 with an International Classification of Diseases (ICD) code for KD or mucocutaneous lymph node syndrome (ICD9 446.1 and ICD10 M30.3) were enrolled.

Data were collected by pediatricians, including demographic information, clinical manifestations, prognosis, laboratory indices, treatment, and echocardiographic findings. The contents of the questionnaire were mildly modified, only with addition of erythema and induration at Bacillus Calmette-Guérin (BCG) sites, sites of valvular regurgitation, the presence of coronary stenosis and thrombosis, and some new laboratory indices including neutrophil count (NEUT), lymphocyte count (LYM), aspartate transaminase (AST), serum sodium (Na), and total bilirubin (TB). After all questionnaires were returned, two senior pediatric cardiologists were responsible for further verification. All eligible cases were entered into the KD database. This study was approved by the institutional review board of Children’s Hospital of Fudan University and requirements for informed consents were waived for this retrospective study.

### Patients and definitions

KD was diagnosed according to the guidelines released by the Japanese Circulation Society.^12^ The diagnostic criteria were as follows: (1) fever persisting for 5 days or longer (including cases in whom the fever has subsided before the 5th day in response to therapy); (2) bilateral conjunctival congestion; (3) polymorphous exanthema; (4) changes of lips and oral cavity: reddening of lips, strawberry tongue, diffuse injection of oral and pharyngeal mucosa; (5) changes of peripheral extremities: reddening of palms and soles in acute phase and membranous desquamation from fingertips in convalescent phase; and (6) acute non-purulent cervical lymphadenopathy. KD was diagnosed in the presence of at least 5 of the 6 principle symptoms. Incomplete KD, referring to KD patients with fewer than 5 principle symptoms, was diagnosed according to the algorithm in AHA guidelines.^1^^,^^13^ Between-hospital transfers during the same episode were identified and repeated cases were excluded. Patients who hospitalized only for further evaluation and treatment of cardiac sequelae (non-acute cases) were also excluded.

IVIG resistance was defined as recurrent or persistent fever (>38°C) after 36 hours of completion of initial IVIG infusion.^1^ Laboratory indices before initial IVIG infusion were recorded, including the highest values of C-reactive protein (CRP), erythrocyte sedimentation rate (ESR), platelet count (PLT), white blood cell count (WBC), creatine kinase-muscle/brain (CK-MB), alanine aminotransferase (ALT), AST, and TB, and the lowest values of hemoglobin (HB), serum albumin (ALB), and Na if laboratory tests were performed more than once before initial IVIG. NEUT and LYM of the same blood test with highest WBC were also recorded.

CAL, defined as coronary dilation or aneurysm, was evaluated using two-dimensional echocardiography. A patient was considered to have CAL if the luminal diameter of a coronary artery was >3.0 mm in children aged <5 years or >4.0 mm in those aged ≥5 years, or when the internal diameter of a segment was ≥1.5 times that of an adjacent segment.^14^ A medium aneurysm was defined as an internal luminal diameter from 4 to 8 mm and a giant aneurysm was defined as an internal luminal diameter ≥8 mm. Coronary measurements of the most severe echocardiography were recorded if the measurement was performed more than once. Coronary thrombosis was assessed using echocardiography or angiography and coronary stenosis was assessed only using coronary angiography.

### Statistical analysis

The incidence of KD was calculated by dividing the number of newly diagnosed KD patients <5 years old who inhabited Shanghai by the resident population of the corresponding age group in Shanghai. Census data were acquired from Shanghai Municipal Center for Disease Control and Prevention.

Data were presented as mean (standard deviation) or median (interquartile range) for continuous variables, and count (percentage) for categorical variables. Continuous variables were compared between groups using unpaired Student’s *t* tests or Mann-Whitney U test. Categorical variables were compared using χ^2^ test or Fisher exact test.

Univariate analysis was performed to explore potential risk factors for CAL, including age of onset, male sex, KD type, the dose and fever days of initial IVIG, and all 13 laboratory indices. Among them, CRP was divided into four groups based on quartiles due to the presence of truncated values. Multivariate logistic regressions were performed to identify risk factors independently associated with CAL.

## RESULTS

### Demographic data and prognosis

Fifty hospitals (100% response rate) returned the questionnaires by the deadline, with 4,533 cases reported. Among them, 37 non-acute cases and 44 repeated cases were excluded. Ultimately, 4,452 cases were enrolled, of which 2,824 (63.4%) were male and 1,628 (36.6%) were female. Male-to-female ratio was 1.7:1.

Sixty patients (1.3%) recurred during this study period, of which 54 (1.2%) relapsed for the first time, 5 (0.1%) for the second, and 1 for the third. The average interval of the first, second, and third recurrence was 13.7 months (range: 0.5–60 months), 8.4 months (range: 1–21 months) and 32 months, respectively. No death was found in this survey.

### Incidence of KD

**Figure 1.**
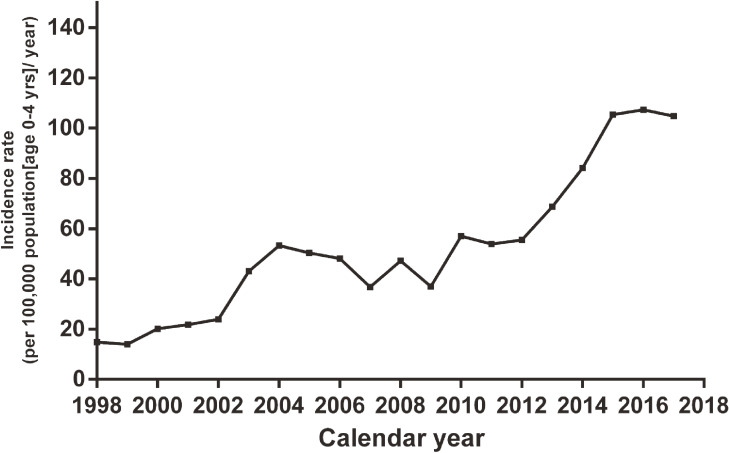
Incidence rate of Kawasaki disease in Shanghai, China from 1998 through 2017.

The incidence of KD was 68.8–107.3 per 100,000 children aged <5 years from 2013 to 2017, with an average of 94.7 (112.7 for males and 75.4 for females; Table 1). It had increased substantially from 2013 to 2015, but remained stable in 2016 and 2017. Generally, the incidence of KD in Shanghai was on the rise since our first survey in 1998 (Figure 1).

**Table 1.** Incidence of Kawasaki Disease in Shanghai, China, from 2013 through 2017 (per 100,000 children younger than 5 years)

Year	Males			Females			Total		
		
	Number of KD cases	Total population	Annual incidence (/100,000)	Number of KD cases	Total population	Annual incidence (/100,000)	Number of KD cases	Total population	Annual incidence (/100,000)
2013	240	256,338	93.6	103	241,937	42.6	343	498,275	68.8
2014	247	244,986	100.8	154	231,271	66.6	401	476,257	84.2
2015	321	262,719	122.2	216	246,978	87.5	537	509,697	105.4
2016	324	274,484	118.0	247	257,505	95.9	571	531,989	107.3
2017	370	293,919	125.9	225	274,904	81.8	595	568,823	104.6
Total	1,502	1,332,446	112.7	945	1,252,595	75.4	2,447	2,585,041	94.7

KD, Kawasaki disease.

### Age of onset

**Figure 2.**
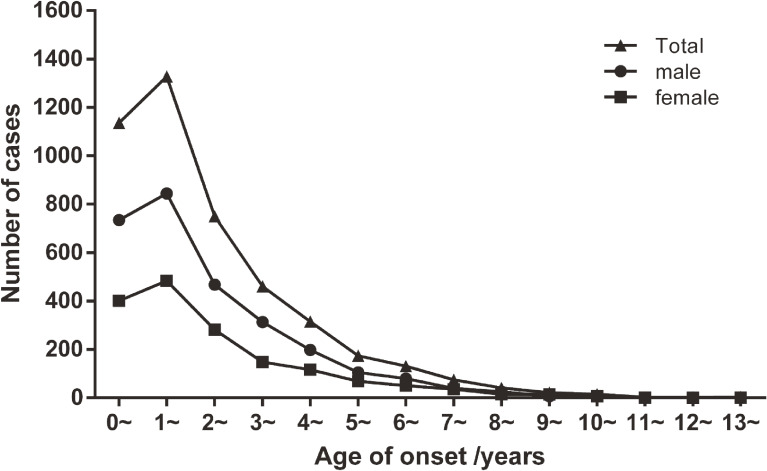
Age distribution of onset of Kawasaki disease in Shanghai, China from 2013 through 2017.

Age at onset of KD ranged from 15 days to 14.0 years (median: 1.8 years), peaking at 1 year of age. A total of 464 cases (10.4%) occurred before 6 months old, 1,133 cases (25.4%) before 1 year old, 2,460 cases (55.3%) before 2 years old, and 3,985 cases (89.5%) before 5 years old. Only one patient, a 15-day-old boy, was a neonate. Males and females shared similar age distribution with the total population (Figure 2).

### Seasonal distribution

**Figure 3.**
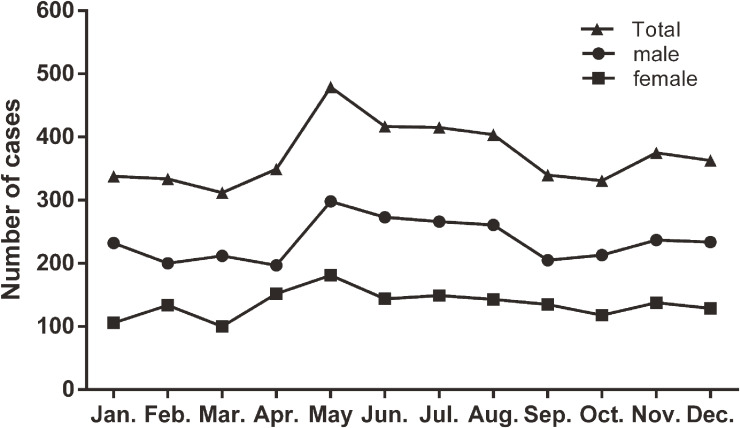
Seasonal distribution of Kawasaki disease in Shanghai, China from 2013 through 2017.

Similar to our previous surveys, KD occurred more frequently in spring (March to May; 1,137 cases, 25.5%) and summer (June to August; 1,235 cases, 27.7%), as compared with autumn (September to November; 1,045 cases, 23.5%) and winter (December to February; 1,035 cases, 23.2%). There was a clear peak in May, which fell to baseline in September after maintaining for 3 months (Figure 3). There were 1,713 cases (38.5%) occurring between May and August.

### Clinical manifestations

The most common manifestation was fever persisting for 5 days or longer (4,304 cases, 96.7%), followed by bilateral conjunctival congestion (3,933 cases, 88.3%), changes in the lips and oral cavity (3,757 cases, 84.4%), polymorphous exanthema (3,502 cases, 78.7%), cervical lymphadenopathy (3,256 cases, 73.1%), and changes in peripheral extremities (2,221 cases, 49.9%). Crissum desquamate (667 cases, 15.0%), and erythema and induration at BCG sites (191 cases, 4.3%) occurred less frequently. BCG reaction occurred more frequently in children under 1 year old (113/1,131 cases, 10.0%) than in those over 1 year old (78/3,321 cases, 2.3%; OR 4.62; 95% confidence interval [CI], 3.43–6.21; *P* < 0.001).

Among the patients, 1,564 (35.1%) presented as incomplete KD, of which 1,020 (65.2%) had four principal signs, 352 (22.5%) had three, and 192 (12.3%) had two. Manifestations of other systems mainly included the involvement of respiratory system (1,291 cases, 29.0%), digestive system (681 cases, 15.3%), hematologic system (159 cases, 3.6%), urinary system (99 cases, 2.2%), skin (71 cases, 1.6%), joint (13 cases, 0.3%), and nervous system (33 cases, 0.7%).

### Treatment

IVIG was administrated to 4,320 cases (97.0%; 6.9 ± 3.4 days, range: 2–55 days), including regimens of 2 g/kg once (2,232 cases, 51.7%), 1 g/kg for two consecutive days (1,671 cases, 38.7%), 1 g/kg once (394 cases, 9.1%) and irregular usage (23 cases, 0.5%).

However, 362 (8.4%) patients failed to respond to the initial treatment. For secondary treatment (354 patients in total because of 8 patients missing further treatment information), a second dose of IVIG (314 cases, 88.7%) was administered most, followed by steroids (41 cases, 11.6%; 1–2 mg/kg, 68.3%) and infliximab (2 cases, 0.6%). Seventy-seven patients (21.8%) were not sensitive to secondary treatment. For third-line or subsequent treatment, steroids (63 cases, 81.8%; ≥10 mg/kg, 55.6%) were administered most, followed by IVIG (28 cases, 36.4%) and infliximab (3 cases, 3.9%). Fever eventually resolved in all patients.

### Cardiovascular abnormalities

A total of 4,447 patients (99.9%) underwent echocardiography within 1 month of onset, of which 406 (9.1%) developed CAL. Except for 26 cases missing coronary measurements, the remaining 380 cases included 231 (5.2%) of dilation and small aneurysms, 118 (2.7%) of medium aneurysms, and 31 (0.7%) of giant aneurysms. Furthermore, the left main coronary artery (LMCA; 287 cases, 75.5%) was the most commonly involved, followed by the right coronary artery (RCA; 187 cases, 49.2%), the left anterior descending artery (LAD; 150 cases, 39.5%), and the left circumflex artery (LCX; 28 cases, 7.4%). Two hundred and ten cases (55.3%) involved one site, 89 cases (23.4%) involved two sites, 60 cases (15.8%) involved three sites, and 21 cases (5.5%) involved four sites simultaneously.

Nine (0.2%) of 4,447 patients had coronary thrombosis, including the involvement of RCA in 6 cases, LMCA in 3 cases, LAD in 2 cases, and LCX in 2 cases. Five of the 9 patients had giant aneurysms and 4 had moderate aneurysms. Coronary stenosis occurred in 14 (0.3%) of 4,447 patients, including the involvement of RCA in 9 cases, LMCA in 7 cases, LAD in 7 cases, and LCX in 1 case. Three patients had severe stenosis, all accompanied with giant aneurysms.

Other cardiovascular abnormalities included cardiomegaly (402 cases, 9.0%), cardiac dysfunction (19 cases, 0.4%), valvular regurgitation (653 cases, 14.7%), and pericardial effusion (408 cases, 9.2%). More specifically, 638 patients (97.7%) had mitral regurgitation (630 mild and 8 moderate cases) and 16 patients (2.5%) had moderate tricuspid regurgitation.

### Risk factors for coronary artery lesions

The laboratory and clinical findings of KD patients with and without CAL were summarized in Table 2. Compared with the none-CAL group, the CAL group had higher levels of CRP, ESR, PLT, and LYM, and lower levels of HB, ALB, and CK-MB. Male sex, incomplete KD, and IVIG resistance were more common in the CAL group. The dose and fever days of initial IVIG also differed between the two groups. WBC, NEUT, ALT, AST, Na, TB, and age of onset were not significantly different.

**Table 2.** Univariate analysis of relevant factors for coronary artery lesions

Variables	Total cases (CAL/none CAL)	CAL	None CAL	*P*
CRP ≥105 mg/L, *n* (%)^a^	387/4,022	118 (30.5)	1,004 (25.0)	0.017
ESR, mm/h, mean (SD)	358/3,823	73.9 (34.1)	70.3 (31.1)	0.041
PLT, 10^12^/L, mean (SD)	387/4,018	465.5 (206.7)	393.7 (163.9)	<0.001
WBC, 10^9^/L, mean (SD)	388/4,027	15.0 (7.4)	14.7 (6.1)	0.679
NEUT, 10^9^/L, mean (SD)	385/3,928	9.8 (7.0)	9.5 (5.5)	0.310
LYM, 10^9^/L, mean (SD)	377/3,883	3.9 (2.1)	3.7 (2.0)	0.032
HB, g/L, mean (SD)	384/4,012	106.4 (14.2)	108.8 (12.4)	<0.001
ALB, g/L, mean (SD)	374/3,902	33.7 (4.5)	35.2 (4.2)	<0.001
ALT, U/L, median (Q1, Q3)	376/3,956	25.0 (13.0, 50.5)	22.0 (12.0, 51.0)	0.175
AST, U/L, median (Q1, Q3)	375/3,942	27.0 (18.0, 42.5)	26.0 (18.0, 39.0)	0.277
CK-MB, IU/L, median (Q1, Q3)	351/3,686	16.0 (12.0, 21.0)	18.0 (13.5, 24.0)	<0.001
Na, mmol/L, mean (SD)	366/3,845	136.6 (3.9)	136.2 (5.5)	0.171
TB, µmol/L, median (Q1, Q3)	366/3,868	6.1 (3.9, 10.0)	6.1 (4.1, 9.4)	0.688
Age of onset, years, median (Q1, Q3)	406/4,041	1.8 (0.9, 3.5)	1.8 (1.0, 3.2)	0.611
Male, *n* (%)	406/4,041	291 (71.7)	2,530 (62.6)	<0.001
Incomplete KD, *n* (%)	406/4,041	181 (44.6)	1,381 (34.2)	<0.001
IVIG resistance, *n* (%)	385/3,931	63 (16.4)	299 (7.6)	<0.001
Fever days at initial IVIG, *n* (%)	385/3,931			<0.001
≤4 days		45 (11.7)	389 (9.9)	
5–10 days		253 (65.7)	3,328 (84.7)	
>10 days		87 (22.6)	214 (5.4)	
Initial IVIG treatment dose, *n* (%)	385/3,931			<0.001
2 g/kg × 1		197 (51.2)	2,034 (51.7)	
1 g/kg × 2		141 (36.6)	1,527 (38.8)	
1 g/kg × 1		35 (9.1)	359 (9.1)	
Irregular usage		12 (3.1)	11 (0.3)	

ALB, serum albumin; ALT, alanine aminotransferase; AST, aspartate transaminase; CAL, coronary artery lesions; CK-MB, creatine kinase-muscle/brain; CRP, C-reactive protein; ESR, erythrocyte sedimentation rate; HB, hemoglobin; IVIG, intravenous immunoglobulin; KD, Kawasaki disease; LYM: lymphocyte count; Na, serum sodium; NEUT, neutrophil count; PLT, blood platelet count; Q1, quartile 1; Q3, quartile 3; SD, standard deviations; TB, total bilirubin; WBC, white blood cell count.

^a^CRP was converted to a dichotomous variable by the upper quartile due to the presence of truncated values.

Therefore, 12 significant variables from the univariate analysis, including CRP, ESR, PLT, LYM, HB, ALB, CK-MB, male sex, incomplete KD, the dose and fever days of initial IVIG, and IVIG resistance, were included in the multivariate analysis to identify risk factors for CAL. It was shown that a higher platelet level, a lower albumin level, male sex, incomplete KD, IVIG resistance, and receiving initial IVIG within 4 days or over 10 days, were independent risk factors for CAL (Table 3).

**Table 3.** Multivariate logistic regression analysis of risk factors for coronary artery lesions (3,627 cases)

Variables	Odds Ratio	95% Confidence Interval	*P*
PLT, 10^12^/L	1.002	1.001–1.003	<0.001
ALB, g/L	0.93	0.90–0.95	<0.001
Male sex	1.64	1.24–2.15	<0.001
Incomplete KD	1.63	1.26–2.09	<0.001
IVIG resistance	2.44	1.72–3.46	<0.001
Fever days at initial IVIG			<0.001
≤4 days	1.62	1.11–2.44	0.014
5–10 days	Ref.	Ref.	
>10 days	4.44	3.14–6.28	<0.001

ALB, serum albumin; IVIG, intravenous immunoglobulin; KD, Kawasaki disease; PLT, blood platelet count.

## DISCUSSION

The incidence of KD in Shanghai was on the rise since 1998, similar to the other Asian countries and regions.^3^^–^^5^^,^^7^ The highest ever record in Shanghai was 107.3 per 100,000 children aged <5 years in 2016, which was still much lower than that in Japan in 2015^3^ (330.2 per 100,000 children aged <5 years) and in South Korea in 2013^4^ (194.9 per 100,000 children aged <5 years), but was for the first time higher than that in Taiwan in 2010^5^ (82.8 per 100,000 children aged <5 years). The incidence of KD in Shanghai remained stable in 2016 and 2017. It requires continuous surveillance, whether or not a 3-year plateau period implies no further increase.

Compared with the high prevalence of fever and five principal symptoms, crissum desquamate (15.0%) and erythema and induration at BCG site (4.3%) occurred less frequently. Not all patients had been checked for BCG site, which might be a reason for the lower rate of BCG site reaction in our study. Changes at the BCG site is increasingly recognized as a specific diagnostic tool for KD in Japan, especially in infants with incomplete KD.^15^^,^^16^ In our study, we also found that BCG site reaction was more prevalent in infants (59.2% for age <1 year). In addition, of 191 patients with positive BCG site reaction, 151 (79.1%) had polymorphous exanthema whereas 40 (20.9%) did not have. Considering the high specificity of BCG reaction in KD patients, we suggest that BCG reaction be added as a supplementary criterion for polymorphous exanthema when diagnosing KD.

The proportion of IVIG administration increased from 71.8% to 97.0% since our first survey in 1998.^6^^,^^11^^,^^17^ In addition, 90.3% of patients received 2 g/kg IVIG as initial treatment and 93.0% received IVIG within 10 days, indicating the standard treatment has been universally adopted in Shanghai. The proportion of IVIG resistance reported so far in Shanghai was 4.9% in 2008–2012^6^ and 8.4% in 2013–2017. The only two reports were not enough to show the trend in our view. Therefore, continuous surveillance is needed. However, the proportion of IVIG resistance was lower than that in Japan^3^ (17.8%), South Korea^4^ (11.8%), and other reported countries (16.3% in the United States,^18^ 9–14% in Canada,^19^ 16.5% in Australia,^20^ 26.8% in German,^21^ and 23.1% in the Netherlands^22^), which may partially be attributed to genetic factors. In addition, studies have shown that administration of IVIG with chemical modifications (such as β-propiolactone and enzyme digestion) led to a higher incidence of IVIG nonresponse than with other manufacturing techniques.^23^^,^^24^ The IVIG used in Shanghai were all pH4 and prepared with acidification rather than chemical modifications, which may also explain the lower incidence of IVIG nonresponse. Given that the mechanism of IVIG action and IVIG resistance remains unclear, it is really difficult to explain the reasons for these discrepancies. More genetic and basic research is needed to clarify it.

Fortunately, the proportion of CAL has substantially decreased from 19.8% to 9.1% since 1998,^11^ possibly attributed to the increasing proportion of IVIG administration and early treatment during the acute phase. Similarly, the proportion of coronary dilation and aneurysm showed a decreasing trend in Japan^3^^,^^25^ (from 10.0% to 6.5%, 2007–2016) and in South Korea^4^^,^^26^ (from 18.5% to 12.5%, 2006–2014). However, the proportion of both medium aneurysms and giant aneurysms lack a significant decrease in Shanghai. In this survey, severe coronary stenosis (all 3 cases) and thrombosis (5 of 9 cases) mainly occurred on the basis of giant aneurysms, increasing the risk of myocardial ischemia and sudden death. Therefore, further prevention of the development of giant aneurysms remains the top priority in the future.

Previous frequently reported risk factors for CAL, such as a higher platelet level,^27^^,^^28^ a lower albumin level,^28^^,^^29^ male sex,^6^^,^^28^^–^^30^ IVIG resistance^6^^,^^29^^,^^31^ and receiving initial IVIG beyond 10 days,^6^^,^^27^^,^^28^^,^^32^ were also demonstrated in our findings. As for incomplete KD, study results were contradictory even in multivariate analyses. One study showed that incomplete KD was more common in the CAL group due to delayed diagnosis rather than the low number of presenting symptoms.^33^ However, consistent with other two studies,^31^^,^^34^ our study identified incomplete KD itself as an independent risk factor for CAL, despite the presence of delayed IVIG treatment.

The last risk factor, receiving initial IVIG within 4 days, was identified beyond expectations. Nomura et al reported that KD patients treated with IVIG before 5 days had a higher incidence of aneurysms than those treated at the fifth day or after (15.2% vs 1.3%; *P* = 0.004).^35^ We also found that patients treated within 4 days had a higher incidence of IVIG resistance (21.4% vs 9.1%; *P* < 0.001), a younger age of onset (1.4 vs 1.8 years; *P* < 0.001), higher levels of CRP (71 vs 62 mg/L; *P* = 0.001), ALT (28 vs 22 U/L; *P* < 0.001), AST (29 vs 26 U/L; *P* < 0.001) and TB (7.3 vs 6.0 µmol/L; *P* < 0.001), and lower levels of Na (135.7 vs 136.2 mmol/L; *P* < 0.001). Considering these, although infusion of IVIG within 4 days was identified as an independent risk factor for CAL in our analysis, it should be interpreted as a sign of severity of KD, which is consistent with Nomura’s views.^35^ Therefore, KD patients diagnosed within 4 days should be treated as early as possible, and may need more aggressive treatment (such as combination with steroids or infliximab). Further prospective studies are needed to confirm this.

There were a couple of limitations in our current study. First, Japanese diagnostic criteria for CAL could lead to underdiagnosis due to the failure of taking patients’ size into account.^36^ Z score, a normalization of coronary dimensions for body surface area, maybe more suitable to evaluate CAL.^37^^,^^38^ Since Z score has not been widely implemented in Shanghai, we still use coronary diameters to evaluate CAL in this survey and the proportion of CAL could be underestimated, although it is encouraging to see its decline in the current survey. Second, epidemiological surveys in Shanghai could to some extent but not completely reflect epidemiologic features of KD in China. We hope to conduct a nationwide sample survey in the future.

In conclusion, the incidence of KD in Shanghai has substantially increased over 20 years. Although the occurrence of CAL has substantially decreased, the proportion of giant aneurysms has remained the same. Higher platelet levels, lower albumin levels, male sex, incomplete KD, IVIG resistance, and receiving initial IVIG within 4 days or beyond 10 days, were independent risk factors for CAL. Continuous epidemiological investigations are still necessary.
